# Severe, Symptomatic Hypercalcemia Secondary to PTH-secreting Pancreatoblastoma

**DOI:** 10.1210/jcemcr/luae217

**Published:** 2024-11-28

**Authors:** Anand D Gandhi, James D McCallum, Jonathan S Fisher

**Affiliations:** Division of Diabetes and Endocrinology, Scripps Clinic, La Jolla, CA 92037, USA; Division of Diabetes and Endocrinology, Scripps Clinic, La Jolla, CA 92037, USA; Division of Organ Transplantation, Scripps Green Hospital, La Jolla, CA 92037, USA

**Keywords:** hypercalcemia, hyperparathyroidism, pancreatoblastoma, parathyroid hormone (PTH)

## Abstract

Hypercalcemia may be induced by a variety of etiologies, most commonly primary hyperparathyroidism. Although primary hyperparathyroidism represents a relatively common endocrinological disorder, ectopic PTH secretion is a rare entity that is less well described in literature. We describe the first case to our knowledge of severe, symptomatic hypercalcemia found to be secondary to a PTH-secreting pancreatoblastoma. The patient initially presented with fatigue and progressive upper extremity intermittent muscular twitching. He was found to have biochemical evidence of primary hyperparathyroidism. A computed tomography scan of the neck and a sestamibi nuclear scan failed to definitively demonstrate a parathyroid adenoma or hyperplasia and bilateral surgical parathyroid exploration was unrevealing for any pathology. Abdominal imaging via computed tomography was obtained for evaluation of progressive postoperative epigastric pain, and the patient was found to have a retroperitoneal mass that, after biopsy, was diagnostic for a pancreatoblastoma. This mass was resected resulting in a fall in intraoperative PTH values and subsequent postoperative hypocalcemia secondary to hungry bone syndrome. Upon follow-up, the patient's parathyroid function recovered and doses of supplemental calcium and vitamin D could be tapered. Ectopic PTH-secreting masses represent a rare entity but should be considered in individuals with unclear etiology of recalcitrant primary hyperparathyroidism.

## Introduction

Hypercalcemia is a common clinical condition seen in approximately 1% of the general population. More than 90% of cases of hypercalcemia can be attributed to either primary hyperparathyroidism or hypercalcemia of malignancy [1]. Primary hyperparathyroidism is characterized by excess secretion of PTH with respect to serum calcium concentration. The most common etiology is a single parathyroid adenoma, accounting for up to 90% of cases of primary hyperparathyroidism [2]. Pathology localized to the parathyroid glands including the aforementioned parathyroid adenoma, multiglandular hyperplasia, and parathyroid carcinoma almost exclusively make up the etiology of this disease process. Hypercalcemia of malignancy from outside the parathyroid glands is most commonly caused by tumor production of PTH-related peptide. Although typically produced by squamous cell cancers (including lung, head, neck, breast, bladder, and ovarian malignancies), it can also be seen with lymphomas and even in patients with T-cell leukemia [3]. Nonparathyroid tumors with ectopic PTH production are an extremely rare cause of primary hyperparathyroidism, with very few cases being reported in literature [4, 5]. We report the first case to our knowledge of a patient presenting with severe, symptomatic hypercalcemia found to be caused by an ectopic PTH-producing pancreatoblastoma.

## Case Presentation

A 22-year-old male with a history of vitamin D deficiency and family history of familial adenomatous polyposis (FAP) presented to the emergency department with intermittent upper extremity muscular twitching and was found to have elevated serum calcium (15.4 mg/dL [3.85 mmol/L]; reference range [RR], 8.4-10.2 mg/dL [2.1-2.6 mmol/L]) alongside elevated intact PTH (463 pg/mL [49.1 pmol/L]; RR, 22.4-88.2 pg/mL [2.40-9.40 pmol/L]) and mild prerenal azotemia. The patient was admitted for further management. Other pertinent laboratory values obtained included 25-OH vitamin D (14.6 ng/mL [36.5 nmol/L]; RR, 30.0-90.0 ng/mL [75-225 nmol/L]), 1,25-dihydroxy vitamin D (112.0 pg/mL [269 pmol/L]; RR, 19.9-79.3 pg/mL [48-190 pmol/L]), 24-hour urine calcium (339 mg/24 hours [0.008 mmol/24 hours; RR, 0-300 mg/24 hours [0-0.007 mmol/24 hours), and PTH-related protein (10 pg/mL [1.25 pmol/L]; RR, <20 pg/mL [<2.5 pmol/L]). He was treated with aggressive IV fluid replacement and 1 dose of zolendronic acid 4 mg IV. Computed tomography (CT) of the neck and larynx with and without contrast demonstrated a possible but not conclusive right parathyroid adenoma. He was discharged shortly thereafter with improvement in symptoms and calcium down to 11.1 mg/dL (2.8 mmol/L); RR, 8.4-10.2 mg/dL (2.1-2.6 mmol/L).

## Diagnostic Assessment

As an outpatient, the patient underwent a sestamibi scan, which did not show any evidence of parathyroid adenoma or ectopic parathyroid tissue in the neck or mediastinum. His serum calcium uptrended again and he was started on cinacalcet 30 mg daily. The cinacalcet provided transient reduction in serum calcium; however, it still remained within hypercalcemic range, and within 2 weeks began uptrending again.

**Figure 1.**
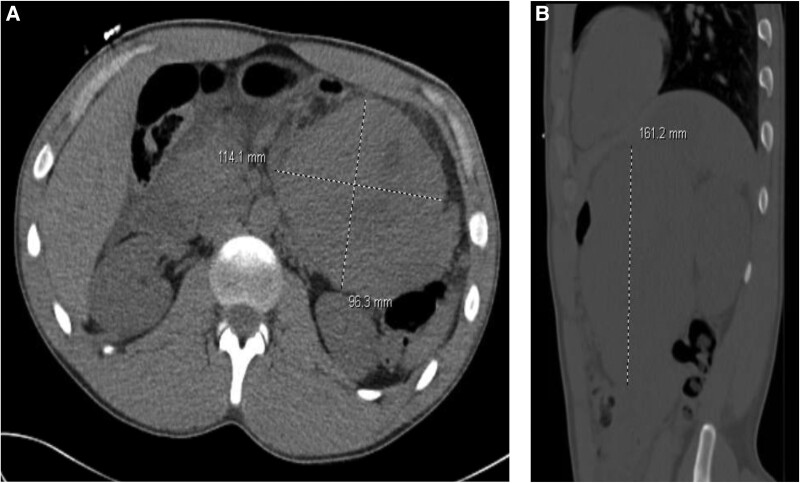
CT of the abdomen and pelvis without IV contrast demonstrating large left retroperitoneal mass inseparable from the pancreatic body and tail. Mass appears to be generally solid with slight hypodensity seen centrally. Mass compresses the stomach, displaced anteriorly. (A) Transverse view of retroperitoneal mass. (B) Sagittal view of retroperitoneal mass.

Because of recalcitrant hypercalcemia with persistent suspicion of a parathyroid lesion, he was admitted for bilateral neck exploration. Two parathyroid glands were excised with surgical pathology not demonstrating any adenomatous or hyperplastic lesion. Intraoperative PTH remained elevated. Postoperatively, the patient developed acute abdominal pain. A CT scan of the abdomen and pelvis with IV contrast was obtained that demonstrated a 16-cm left retroperitoneal necrotic mass inseparable from the pancreatic body and tail (Fig. 1). There was evidence of local adenopathy in the retroperitoneum and porta hepatis. The patient underwent endoscopic ultrasound with fine-needle aspiration of the mass, which revealed a pancreatoblastoma. He was discharged with arrangements for close outpatient follow-up for surgical planning.

Four weeks following discharge, he was readmitted for management of worsening hypercalcemia. Throughout the early part of his hospitalization, he received a second dose of zoledronic acid 4 mg IV, 2 daily sequential doses of calcitonin 4 U/kg IM, and denosumab 120 mg subcutaneously resulting in normalization of serum calcium.

He underwent a colonoscopy for operative planning purposes because of a family history of FAP and adenocarcinoma of the colon in his father. His colonoscopy demonstrated >100 small polyps measuring 5 to 10 mm, consistent with FAP; however, without any masses concerning for malignancy, the decision was made to defer prophylactic colectomy.

## Treatment

He underwent subtotal distal pancreatectomy and splenectomy. Surgical pathology confirmed pancreatoblastoma of the distal pancreas with focal lymphovascular invasion and 2/6 positive lymph nodes. Histologically, the neoplasm was composed of small malignant cells in a sheet growth pattern with focal squamoid nests, brisk mitotic activity, and focal tumor necrosis. Immunohistochemical staining was positive for AE1/AE3, CK5, along with diffuse nuclear β-catenin expression.

**Figure 2.**
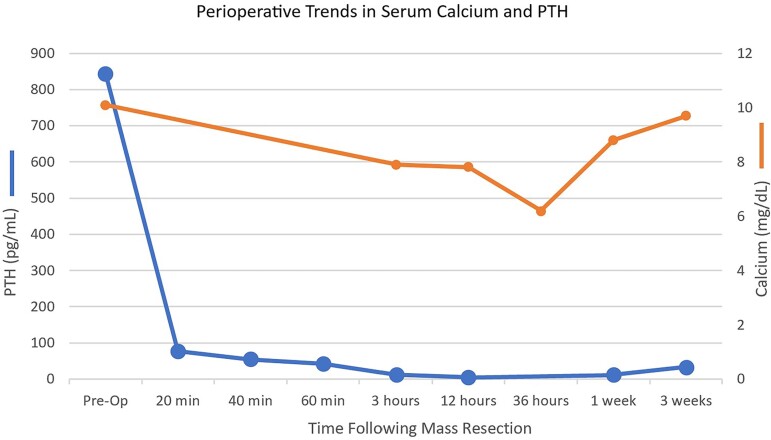
Double line graph showing PTH and calcium levels perioperatively. Evidence of substantial hyperparathyroidism before surgical intervention with rapid downtrend intraoperatively. Noted hypoparathyroidism postoperatively followed by normalization in the following 3 weeks. Calcium remained stable intraoperatively; however, it was met by postoperative decline with normalization within the following week.

Intraoperative PTH level declined rapidly from 843.5 pg/mL (89.4 pmol/L); RR, 22.4-88.2 pg/mL (2.40-9.40 pmol/L) at the start of the procedure to 42.7 pg/mL (4.53 pmol/L); RR, 22.4-88.2 pg/mL (2.40-9.40 pmol/L) 60 minutes after removing the mass (Fig. 2).

His postoperative course was complicated by hypocalcemia to a nadir of 6.2 mg/dL (1.5 mmol/L); RR, 8.4-10.2 mg/dL (2.1-2.6 mmol/L), hypoparathyroidism to a nadir of PTH 5.0 pg/mL (0.53 pmol/L); RR, 22.4-88.2 pg/mL (2.40-9.40 pmol/L), and hyperglycemia. He required aggressive calcium and calcitriol replacement following surgery alongside low-dose correctional insulin therapy for hyperglycemia.

Gradually, over 1 week postoperatively, serum calcium normalized with the patient receiving calcium citrate 800 mg 3 times daily and calcitriol 0.5 mcg daily. At time of discharge, he tolerated appropriate oral intake and did not require further insulin therapy.

## Outcome and Follow-up

Three weeks after discharge, his serum calcium levels and PTH levels had normalized to 9.7 mg/dL (2.4 mmol/L); RR, 8.4-10.2 mg/dL (2.1-2.6 mmol/L) and 33.6 pg/mL (3.6 pmol/L); RR, 22.4-88.2 pg/mL (2.40-9.40 pmol/L), respectively. His calcium supplement dose was lowered to 600 mg twice daily and his calcitriol supplement was discontinued. He described substantially improved appetite and energy levels and reported now being able to carry out his day-to-day activities.

Two months following discharge, the patient presented with a partial small bowel obstruction that resolved with conservative management. Repeat CT of the abdomen/pelvis with IV contrast also demonstrated a new 1.7-cm right hepatic lobe lesion concerning for metastasis. He has established care with an oncologist as an outpatient who recommends combination cisplatin and doxorubicin chemotherapy regimen for adjuvant treatment. He is planning on starting this regimen in the near future.

## Discussion

Hypercalcemia is a common clinical disorder most often the result of primary hyperparathyroidism or humoral hypercalcemia of malignancy [1]. Single parathyroid adenomas or multiglandular hyperplasia are responsible for most causes of primary hyperparathyroidism [2]. Surgical management of this disorder involves imaging localization of possible parathyroid pathology followed by surgical excision. In select cases, bilateral neck exploration may be required in the absence of definitive localization if high suspicion of parathyroid pathology persists. Such intervention exhibits high rates of operative success in high-volume centers; however, in a small minority of instances, biochemical cure is not attained. Although operative failure most often is due to missed pathologic parathyroid glands, ectopic PTH secreting masses have been described in literature as an occult culprit of disease.

Nonparathyroid PTH-secreting neoplasms represent an extremely rare cause of primary hyperparathyroidism with only a few cases reported in literature. Prior reports describe cases of intact PTH production from an ovarian carcinoma [4], neuroendocrine tumor [5], and small cell lung carcinoma [6].

Our patient presented with severe, symptomatic hypercalcemia and was found to have evidence of overt primary hyperparathyroidism. Although imaging evaluation was indeterminate, with an elevated PTH level and a normal PTH-related peptide level, pretest suspicion was sufficiently high to pursue bilateral neck exploration. Despite thorough surgical investigation with excision of 2 glands, no parathyroid pathology was demonstrated and surgical cure was not initially achieved.

It was only after the development of abdominal pain that a large retroperitoneal mass was elucidated through imaging. This mass, following biopsy and immunohistochemical staining, was found to be suggestive of a pancreatoblastoma. Following surgical removal of this mass, the patient's PTH levels fell precipitously, giving credence to the potential paraneoplastic nature of this tumor. Our patient represents to our knowledge the first documented case of a PTH-secreting pancreatoblastoma.

Pancreatoblastomas are rare pancreatic tumors most commonly found in infants and children, at a median age of 5 years [7]. Only 23 cases of pancreatoblastomas have been documented in adults. Just as in our patient, adults with such masses tend to be more symptomatic compared to children and typically display abdominal pain as their initial presenting complaint [7]. Prior cases document frequent invasion into adjacent structures such as the duodenum [8], along with distant or nodal metastasis [9].

The pathogenesis of pancreatoblastomas is unknown. Immunohistochemical staining of our patient's pancreatoblastoma demonstrated positivity for diffuse nuclear β-catenin expression. Molecular studies have demonstrated alterations in the adenomatous polyposis coli (APC)/β-catenin signaling pathway in these masses [10] as well as in familial isolated hyperparathyroidism [11]. Biallelic inactivation of the APC gene has been previously identified in patients with pancreatoblastomas arising in the setting of FAP as well [10]. Via whole blood genetic testing, our patient did demonstrate 1 pathogenic variant of the APC gene with known association to FAP: c.3183_3187del; p.Gln1062Ter. These findings underscore a possible association between pancreatoblastomas and FAP stemming from common aberrations in signal transduction pathways. More research is needed, however, to further characterize this association in detail.

## Learning Points

- Ectopic PTH-secreting masses represent an uncommon etiology of primary hyperparathyroidism
- Pancreatoblastoma is a rare pancreatic tumor with paraneoplastic potential
- Providers should maintain nonparathyroid sources of PTH production in the differential for primary hyperparathyroidism of unknown source

## Contributors

All authors made individual contributions to authorship. A.G. and J.M. were involved in the diagnosis and management of this patient. J.F. was responsible for the patient's surgeries. All authors reviewed and approved the final draft.

## Data Availability

Data sharing not applicable to this article as no datasets were generated or analyzed during the current study.

## Abbreviations

APC: adenomatous polyposis coli
CT: computed tomography
FAP: familial adenomatous polyposis
RR: reference range

## References

[1] Walker MD, Shane E. Hypercalcemia: a review. J Am Med Assoc. 2022;328(16):1624‐1636. 10.1001/jama.2022.18331 36282253

[2] Ruda JM, Hollenbeak CS, Stack BC Jr. A systematic review of the diagnosis and treatment of primary hyperparathyroidism from 1995 to 2003. Otolaryngol Head Neck Surg. 2005;132(3):359‐372. 15746845 10.1016/j.otohns.2004.10.005

[3] Stewart AF . Hypercalcemia associated with cancer. N Engl J Med. 2005;352(4):373‐379. 15673803 10.1056/NEJMcp042806

[4] Nussbaum SR, Gaz RD, Arnold A. Hypercalcemia and ectopic secretion of parathyroid hormone by an ovarian carcinoma with rearrangement of the gene for parathyroid hormone. N Engl J Med. 1990;323(19):1324‐1328. 2215618 10.1056/NEJM199011083231907

[5] Kandil E, Noureldine S, Khalek MA, Daroca P, Friedlander P. Ectopic secretion of parathyroid hormone in a neuroendocrine tumor: a case report and review of the literature. Int J Clin Exp Med. 2011;4(3):234‐240. 21977238 PMC3182517

[6] Yoshimoto K, Yamasaki R, Sakai H, et al Ectopic production of parathyroid hormone by small cell lung cancer in a patient with hypercalcemia. J Clin Endocrinol Metab. 1989;68(5):976‐981. 2541161 10.1210/jcem-68-5-976

[7] Cavallini A, Falconi M, Bortesi L, et al Pancreatoblastoma in adults: a review of the literature. Pancreatology. 2009;9(1-2):73‐80. 19077457 10.1159/000178877

[8] Montemarano H, Lonergan GJ, Bulas DI, Selby DM. Pancreatoblastoma: imaging findings in 10 patients and review of the literature. Radiology. 2000;214(2):476‐482. 10671596 10.1148/radiology.214.2.r00fe36476

[9] Klimstra DS, Wenig BM, Adair CF, Heffess CS. Pancreatoblastoma. A clinicopathologic study and review of the literature. Am J Surg Pathol. 1995;19(12):1371‐1389. 7503360 10.1097/00000478-199512000-00005

[10] Abraham SC, Wu TT, Klimstra DS, et al Distinctive molecular genetic alterations in sporadic and familial adenomatous polyposis-associated pancreatoblastomas: frequent alterations in the APC/β-catenin pathway and chromosome 11p. Am J Pathol. 2001;159(5):1619‐1627. 11696422 10.1016/s0002-9440(10)63008-8 PMC1867075

[11] Cetani F, Pardi E, Borsari S, et al Exome analysis of a large family with familial isolated primary hyperparathyroidism (FIHP) and multiple cancers. Paper presented at: 19th European Congress of Endocrinology; 2017, Lisbon, Portugal, p 348 (Abstract P2–5).

